# Supplementary figures and images for: Transcriptome analysis of Chinese mitten crabs (Eriocheir sinensis) gills in response to ammonia stress
Source: PeerJ. 2024 Jan 16;12:e16786. doi: 10.7717/peerj.16786 (PMC10798153; doi:10.7717/peerj.16786)

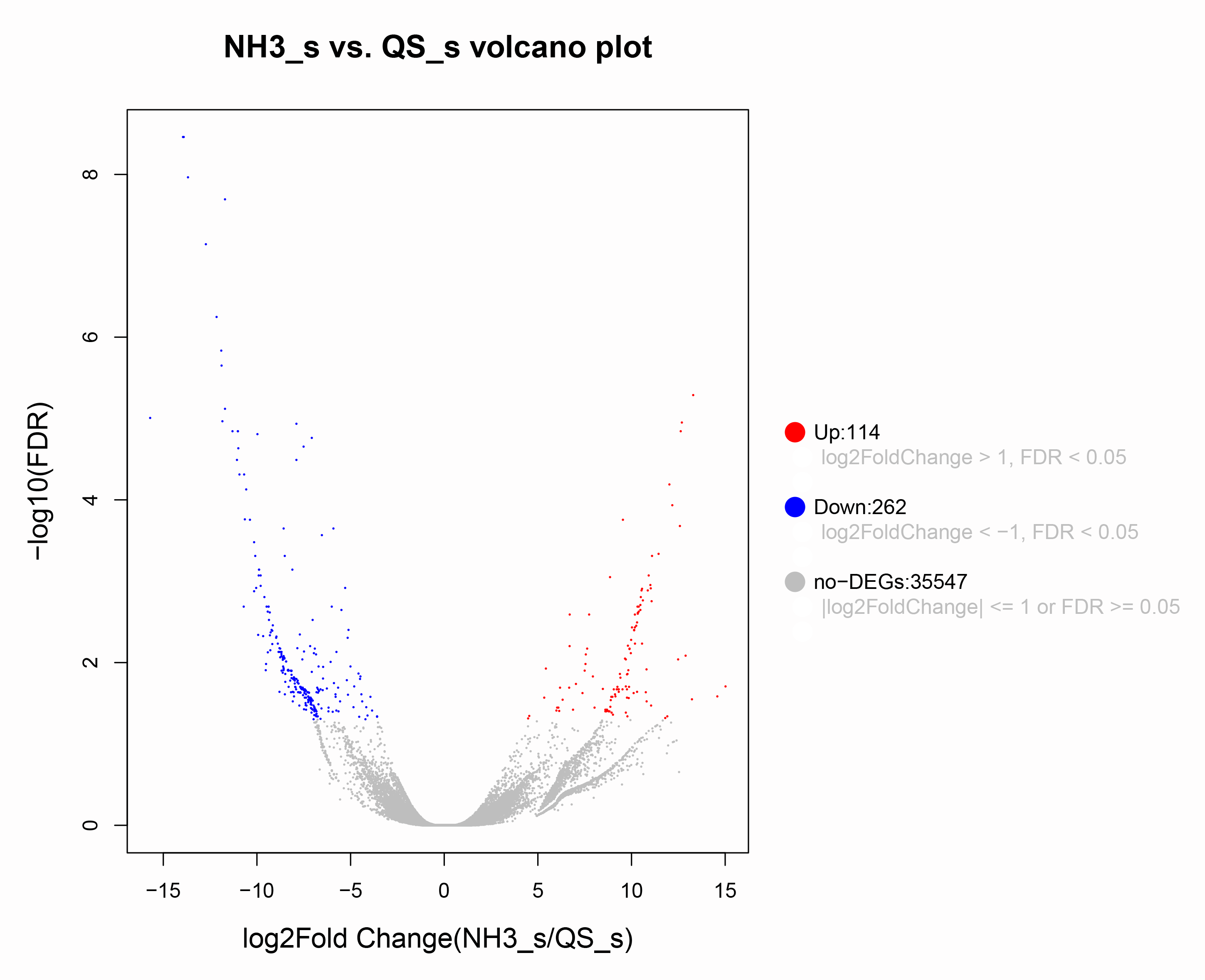

Supplement: Supplemental Information 1 — The blue scatters indicate the down-regulated genes, and the red scatters indicate the up-regulated genes. NH3_S represent ammonia stress group (AG) and QS_S represent control group (CG). [file peerj-12-16786-s001.png]

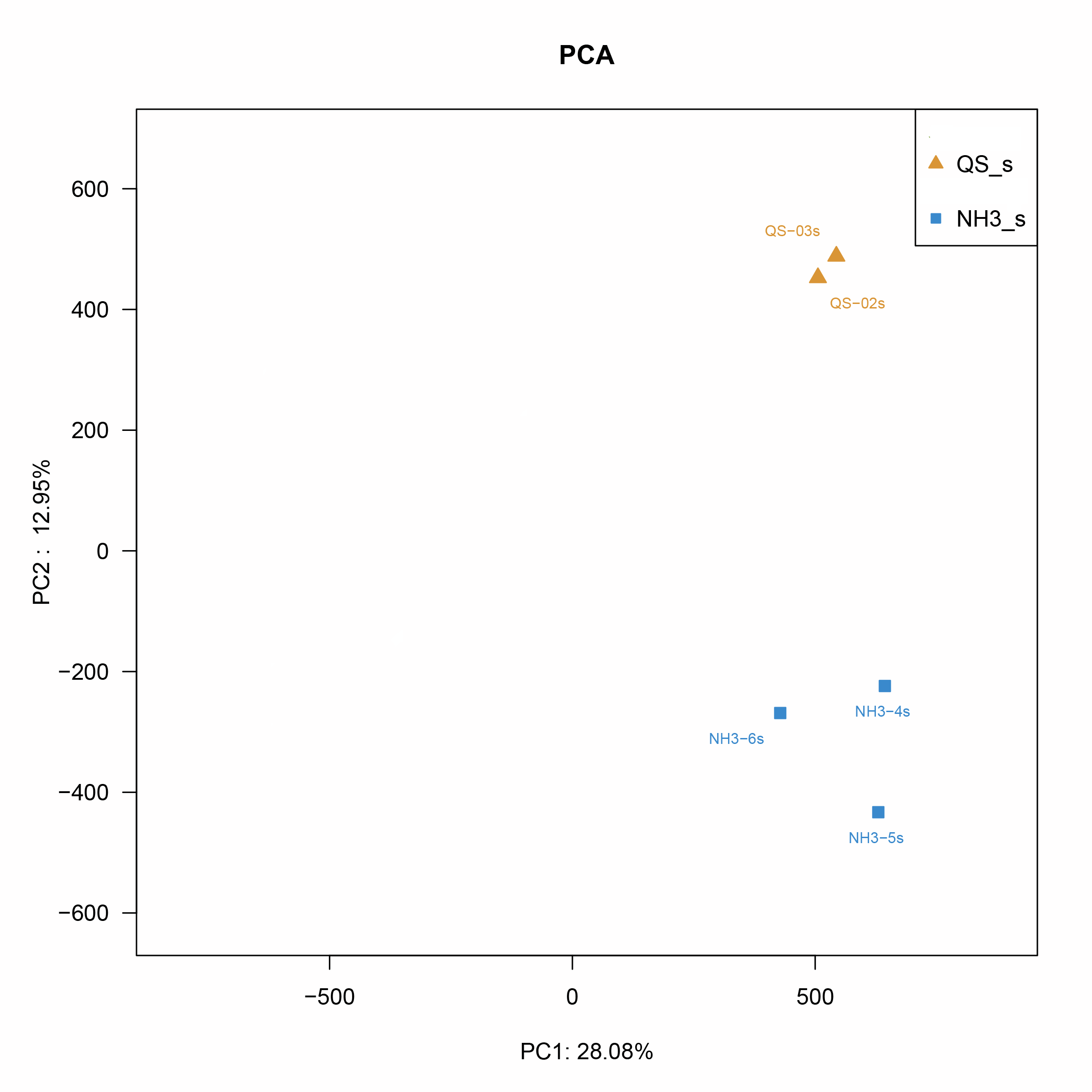

Supplement: Supplemental Information 2 — QS-2s represents the CG-2, QS-3s represents the CG-3, NH4-3 represents the AG-1, NH4-4 represents the AG-2, NH3-5 represents the AG-3. [file peerj-12-16786-s002.png]

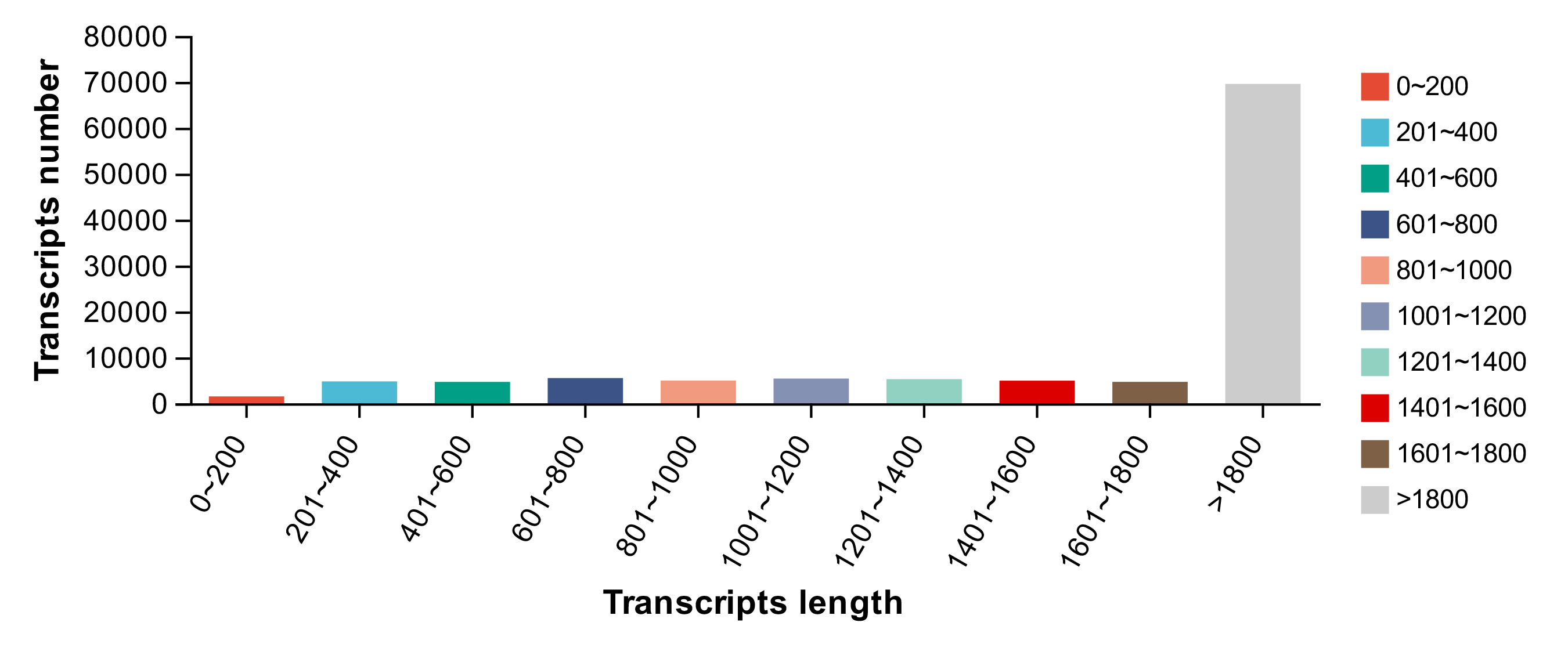

Supplement: Supplemental Information 3 [file peerj-12-16786-s003.png]
