# Supplementary material for: Transcriptome analysis of Chinese mitten crabs (Eriocheir sinensis) gills in response to ammonia stress
Source: PeerJ. 2024 Jan 16;12:e16786. doi: 10.7717/peerj.16786 (PMC10798153; doi:10.7717/peerj.16786)
Supplement: Supplemental Information 4 [file peerj-12-16786-s004.docx]

**Supplemental Table S1：**

**The annotated results of gene and transcripts based on the reference genome**

| Database | Expre_Gene number | | Expre_Transcript number | All_Gene number | All_Transcript number |
| --- | --- | --- | --- | --- | --- |
| Nr | 17551 | 45086 | | 21472 | 59350 |
| Swiss-prot | 12358 | 33572 | | 14158 | 42531 |
| Pfam | 12983 | 32066 | | 14925 | 41428 |
| GO | 11165 | 27842 | | 13174 | 59165 |
| KEGG | 10455 | 28741 | | 11927 | 35849 |
| COG | 14123 | 37911 | | 16269 | 48034 |
