# Supplementary material for: Transcriptome analysis of Chinese mitten crabs (Eriocheir sinensis) gills in response to ammonia stress
Source: PeerJ. 2024 Jan 16;12:e16786. doi: 10.7717/peerj.16786 (PMC10798153; doi:10.7717/peerj.16786)
Supplement: Supplemental Information 5 [file peerj-12-16786-s005.docx]

**Supplemental Table S1：**

**BLAST analysis of non-redundant unigenes against public databases**

| Database | Number of annotated unigenes | Percentage of annotated unigenes (%) |
| --- | --- | --- |
| Nr | 20,648 | 3.12 |
| Swiss-prot | 13,634 | 2.06 |
| String | 14,418 | 2.18 |
| Pfam | 8,927 | 1.35 |
| GO | 13,503 | 2.04 |
| KEGG | 11,362 | 1.72 |
